# Unipolar Electrogram-Guided versus Lesion Size Index-Guided Catheter Ablation in Patients with Paroxysmal Atrial Fibrillation

**DOI:** 10.3390/jcdd9070229

**Published:** 2022-07-18

**Authors:** Guohua Fu, Bin He, Binhao Wang, Mingjun Feng, Xianfeng Du, Jing Liu, Yibo Yu, Fang Gao, Weidong Zhuo, Yi Xu, Yingbo Qi, Huimin Chu

**Affiliations:** 1 Arrhythmia Center, Ningbo First Hospital, Ningbo 315000, China; eagle1002@126.com (G.F.); socrates_he@126.com (B.H.); wangbinhao0504@163.com (B.W.); fmj76@126.com (M.F.); drduxianfeng@126.com (X.D.); nblight6@126.com (J.L.); mubird@foxmail.com (Y.Y.); amy_gf89@163.com (F.G.); zhuoweidong1995@163.com (W.Z.); xuyi2333@zju.edu.cn (Y.X.); qyb19971011@163.com (Y.Q.); 2 Key Laboratory of Precision Medicine for Atherosclerotic Diseases of Zhejiang Province, Ningbo 315000, China

**Keywords:** unipolar electrogram, lesion index, paroxysmal atrial fibrillation, pulmonary vein isolation

## Abstract

Background: This research explores the relationship between the unipolar electrogram (UP-EGM) and lesion size index (LSI) in different regions of continuous circular lesions (CCLs) and to assess the safety and efficacy of UP-EGM-guided versus LSI-guided radiofrequency catheter ablation (RFCA) in patients with paroxysmal atrial fibrillation (PAF). Methods: A total of 120 patients with drug-refractory PAF who underwent index RFCA were scheduled to be consecutively included from March 2020 to April 2021. All the patients were randomly divided 1:1 into two groups: the UP-EGM group and the LSI group. The first-pass PVI rate, acute PVI rate, and the sinus rhythm maintenance rate were compared. Results: A total of 120 patients with PAF were included in the study: the UP-EGM group (*n* = 60) and the LSI group (*n* = 60). All the LSI values in the UP-EGM group were less than those in the corresponding regions in the LSI group (all *p* < 0.001). There were no significant differences in the first-pass PVI rate and acute PVI rate between the two groups. After a mean follow-up period of 11.31 ± 1.70 months, the sinus rhythm maintenance rate in the UP-EGM group was comparable to that in the LSI group (90% vs. 91.7%, *p* = 0.752). Conclusion: UP-EGM-guided and LSI-guided RFCA are both effective and safe in patients with PAF. However, UP-EGM may be more suitable than LSI for guiding individual RFCA.

## 1. Introduction

Pulmonary vein isolation (PVI) is the cornerstone of catheter ablation procedures in patients with paroxysmal atrial fibrillation (PAF). However, long-lasting, continuous, and transmural PVI is still a clinical challenge [1]. The lesion size index (LSI) was defined as a multiparameter index incorporating the power, contact force (CF), impedance, and time, and was found to be a useful guide for PVI. In these studies, constant values of LSI for the anterior (4.5–5.5) and posterior (4.0–4.5) regions of continuous circular lesions (CCLs) around the pulmonary vein ostia were recommended [2,3,4,5,6]. However, the thickness of atrial tissue differs in different regions and individuals. The constant values of LSI, which do not take the variations in the underlying left atrial thickness into account, are the disadvantage of LSI-guided catheter ablation [7,8]. Previous investigations have demonstrated that the elimination of the negative component of the unipolar electrogram (UP-EGM) during radiofrequency (RF) applications reflects transmural lesions and that unipolar signal modification is an effective guide for PVI that can automatically adjust according to the variations in the left atrial thickness [9,10,11,12]. Thus, UP-EGM may be more suitable than LSI to guide individual catheter ablation for PAF. The aim of this study was to explore the relationship between UP-EGM and LSI in different CCL regions and to assess the safety and efficacy of UP-EGM-guided versus LSI-guided RF catheter ablation (RFCA) in patients with PAF.

## 2. Materials and Methods

### 2.1. Study Population

A total of 120 patients with drug-refractory PAF who underwent index RFCA in the Arrhythmia Center of Ningbo First Hospital were scheduled to be consecutively included from March 2020 to April 2021. The exclusion criteria were as follows: (1) age < 18 or >80 years old, (2) left atrium (LA) diameter > 45 mm, (3) the presence of a mechanical mitral valve prosthesis, (4) impaired thyroid function, (5) left ventricular ejection fraction < 40%, (6) contraindication for anticoagulant therapy, (7) current malignancy, (8) prior catheter or surgical AF ablation, and (9) life expectancy < 1 year.

All patients were randomly divided 1:1 into 2 groups: the UP-EGM group and the LSI group. The protocol was approved by the local institutional ethics committees and complied with the Declaration of Helsinki. All participants were fully informed and provided written consent.

### 2.2. Mapping and RF Catheter Ablation

The use of antiarrhythmic drugs (AADs) was stopped five half-lives before the procedure. The absence of a thrombus in the LA was confirmed using transesophageal echocardiogram (TEE) or cardiac CT angiogram one day before the procedure.

**Figure 1:**
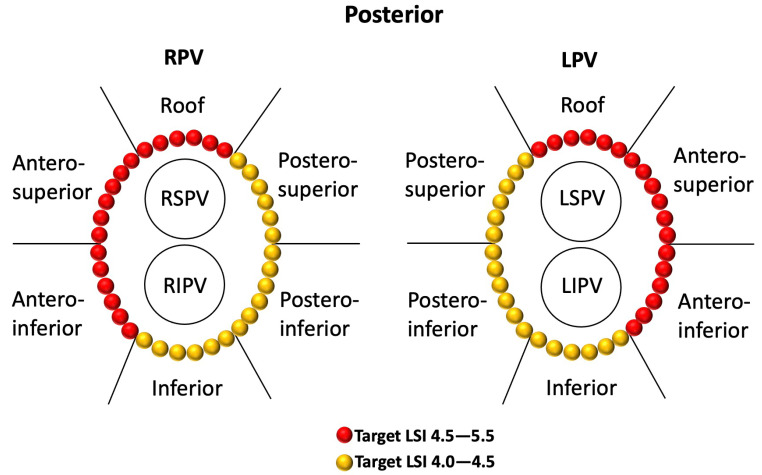
Regional definitions of right and left CCLs and schematic diagrams of target LSIs in different regions of CCLs in the LSI group. The target LSI values were 4.5–5.5 for the roof and anterior walls and 4.0–4.5 for the posterior and inferior walls, respectively. CCLs—continuous circular lesions; LSI—lesion size index.

The patients were placed in the supine position under deep sedation for ablation. One dispersive patch was used during the procedure and placed at the lower back. A deflectable multielectrode catheter was positioned inside the coronary sinus (CS) via the left femoral vein. After double transseptal puncture was performed from the right femoral vein access, anticoagulation with heparin was initiated to maintain a target-activated clotting time of 250–350 s. Through transseptal access, a non-steerable sheath (SL1™, 8.5F, Abbott, MN, USA) and a steerable sheath (Agilis™, 11.5F, Abbott, MN, USA) were placed into the LA. Then, the 20-pole circular mapping catheter (AFocus II™ Double Loop, Abbott, MN, USA) and a 7F unidirectional irrigated tip sensor force ablation catheter (Tacticath™ Quartz Abbott, MN, USA) were advanced into the LA via the above sheaths. The NavX™ Velocity System (Abbott, MN, USA) was used to create a three-dimensional electroanatomic map and intracardiac EGM recordings. The filter settings were 30–500 Hz for the bipolar (BP) EGM and 0.5–180 Hz for the UP-EGM. All CCLs were completed by point-to-point ablation. Both CCLs were divided into six regions, including roof, antero-superior, antero-inferior, inferior, postero-inferior, and postero-superior (Figure 1) [12]. The RF ablation settings used were 40 W/43 °C/25 mL per minute for the roof and anterior walls and 35 W/43 °C/17 mL per minute for the posterior and inferior walls, with a target force of 5–15 g. For the stability of the catheter position, the AutoMark settings for the filter thresholds were as follows: the minimum marker time was 3 s, the marker spacing was 6 mm, and the away time was 5 s. The lesion tag size was 4 mm and the target interlesion distance (ILD) between the two neighboring lesions was 5 mm or less according to the recommendation of a previous study [13,14]. PVI was guided by LSI or UP-EGM. PVI (entrance and exit block) was verified as the absence of any PV or LA potential in the PV antral ablation area using a circular catheter and/or ablation, and a complete bidirectional block was systematically confirmed by pacing (output of 20 mA at a pulse width of 2 ms) for each bipole of the Lasso catheter [1,12]. Thirty minutes after PVI, the complete bidirectional blocks were rechecked for each PV. In the case of PV reconnection, supplemental RF applications were performed for the gaps to reisolate PVs. AF induction attempts were carried out at the end of the procedures. Supplemental RF applications were performed only for the trigger of AF. The cavotricuspid isthmus was ablated only in cases of documented common flutter; LA nests were not targeted for ablation, and no LA linear lesions were deployed.

#### 2.2.1. LSI-Guided PVI

The target LSI values were 4.5–5.5 for the roof and anterior walls and 4.0–4.5 for the posterior and inferior walls, respectively (Figure 1) [3,4,5,6,15]. Once the highest target LSI was reached, the RF application was stopped, and the catheter was moved to an adjacent spot. The ablation time for each point was limited to 30 s.

#### 2.2.2. UP-EGM-Guided PVI

**Figure 2:**
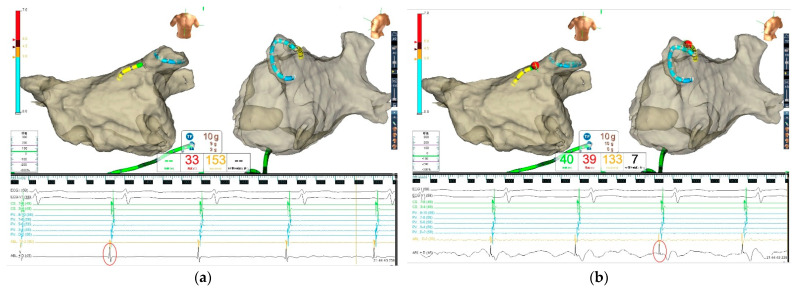
Catheter ablation guided by UP−EGM. Every point in the CCLs was ablated until the UP−EGM turned completely positive and was extended 5 s after elimination of the negative component of the UP−EGM. The LSI value for every ablation point was also recorded for comparison. (**a**) Before ablation, the UP−EGM of the point showed a negative component (in the red circle). (**b**) After ablation, UP−EGMs turned completely positive (in the red circle). UP−EGM—unipolar electrogram; CCLs—continuous circular lesions. The Green, Red and Black Chinese word in Figure 2 indicated Power, Temperature and RF time respectively.

All procedures in this group were performed in sinus rhythm (SR) because accurate analysis of the UP-EGM modification is suboptimal during AF. Therefore, patients underwent cardioversion if they presented with AF at the beginning of or during the procedure. The complete positive UP-EGM was the endpoint for every RF point and was extended 5 s after elimination of the negative component of UP-EGM (Figure 2) [11,12]. The LSI value for every ablation point was also recorded. The ablation time for each point was limited to 30 s if the UP-EGM could not turn completely positive. All the UP-EGMs were recorded in real time with the NavX™ Velocity System at a sweep speed of 200 mm/s during ablation.

### 2.3. Postablation Management and Follow-Up

All patients were discharged within 4 days (limits, 2–4 days). AADs and anticoagulants (novel oral anticoagulant or vitamin K antagonists) were prescribed for 3 months. Subsequently, the AADs were discontinued, and the usage of anticoagulants depended on the CHA_2_DS_2_-VASc score. The patients were followed in the outpatient department at 3-, 6-, and 12-months post-ablation. A 12-lead ECG and continuous 24-h or 7-day Holter monitoring were performed routinely in all patients at each follow-up visit. Atrial tachycardia (AT)/AF recurrence was defined as any episode lasting >30 s (either symptomatic or asymptomatic) subsequent to a 3-month blanking period [1].

### 2.4. Statistical Analysis

Continuous data are presented as the mean ± SD. Dichotomous data are expressed as numbers and percentages. Continuous variables with a nonnormal distribution are shown as the median and interquartile range. Variables were compared using Student’s *t* test, the Mann–Whitney U test, and Fisher’s exact test where appropriate. The Kaplan–Meier estimate was used to compare the rate of freedom from AF between both groups during the 12-month follow-up period. *p* values < 0.05 were considered statistically significant. All statistical analyses were performed in SPSS Statistics 25 (IBM Corporation, Armonk, NY, USA).

## 3. Results

### 3.1. Baseline Characteristics

A total of 120 consecutive patients were enrolled in the study and were randomly divided 1:1 into two groups: the UP-EGM group (*n* = 60) and the LSI group (*n* = 60). The baseline characteristics of the study population are shown in Table 1. There were no significant differences in the baseline characteristics (i.e., age, sex, body mass index, history of AF, CHA2DS2-VASc and HAS-BLED scores, comorbidity, LA diameter, and LVEF) between the groups.

### 3.2. Index AF Ablation Procedure

The data of the index AF ablation procedure for both groups are shown in Table 2. All PVs were isolated with a complete bidirectional block in all patients. In the UP-EGM group, all the recorded UP-EGMs turned completely positive, and four different types of completely positive UP-EGM morphology patterns (R, rR′, Rr′, and M patterns) were recorded as described in our previous study [12]. The procedural time (defined as the time from the puncture to the removal of the sheath), X-ray exposure, ablation time, and ablation energy delivered were significantly lower in the UP-EGM group than in the LSI group. The difference in the mean CF and the mean impedance decrease (Ω) (the range of the baseline impedance was from 120 Ω to 160 Ω) did not reach statistical significance between the two groups.

Cavotricuspid isthmus ablation was performed on six (10%) and five (8.3%) patients in the UP-EGM group and LSI group, respectively. One patient (1.7%) each in both groups underwent superior vena cava isolation. The rate of additional ablation was comparable between the two groups.

One patient (1.7%) in the UP-EGM group had a pseudoaneurysm, and one patient (1.7%) in the LSI group developed reactive pneumonia. No esophageal injury, phrenic nerve injury, cardiac tamponade, or stroke occurred in either group.

### 3.3. The First-Pass PVI Rate

In the UP-EGM group, 11 patients (18.3%) required targeting of the PV carina despite the creation of a circular lesion around the ipsilateral PV ostia, exhibiting completely positive UP-EGMs. Thirty minutes after PVI, six patients (10%) presented with PV reconnection. Therefore, the first-pass PVI rate was 90% (54/60). The UP-EGM morphology of the gaps demonstrated a negative component, and the PVs were reisolated in these patients by targeting those reversed UP-EGMs of the gaps.

In the LSI group, 12 patients (20%) required targeting of the PV carina after the CCLs were completed. After 30 min of waiting time, five patients (8.3%) presented with PV reconnection, resulting in a first-pass PVI rate of 91.7% (55/60). In these five patients, the PVs were reisolated by targeting the earliest PV potentials recorded by the Lasso catheter. The mean LSI value of these supplementary ablation points was 4.90 ± 0.27.

There were no significant differences in the first-pass PVI rate and acute PVI rate between the two groups.

### 3.4. LSI Values in Different Regions of Continuous Circular Lesions (CCLs)

The total ablation points for the UP-EGM group and the LSI group were 5164 (average: 86.1 ± 12.5) and 5383 (average: 89.7 ± 10.6), respectively (*p* = 0.087). The mean LSI values in the different regions of the CCLs are shown in Table 3.

**Figure 3:**
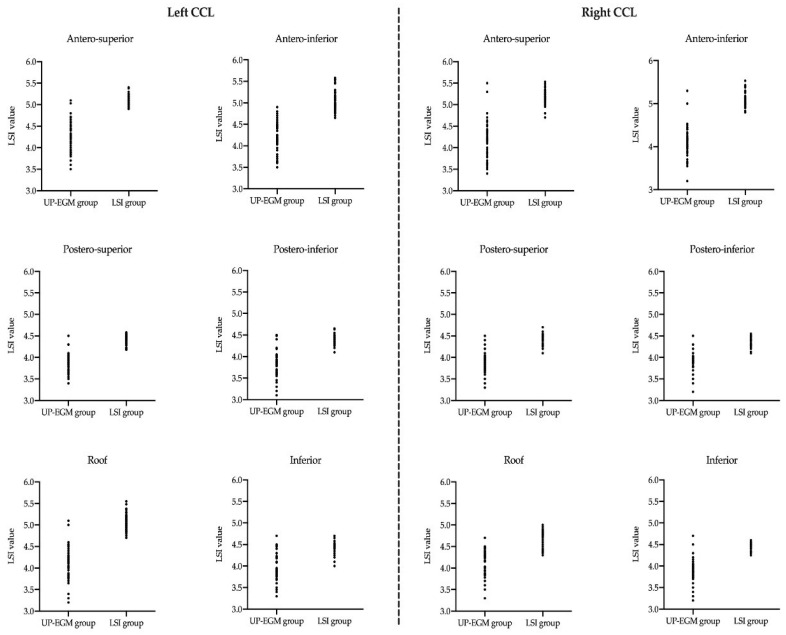
The scatter diagram of the average LSI values in different regions of CCLs in both groups. (**Left**): six regions in left CCL. (**Right**): six regions in right CCL. The variation in the LSI values in the UP-EGM group was significantly greater than that in the LSI group in all CCL regions. LSI—lesion size index; UP-EGM—unipolar electrogram; CCLs—continuous circular lesions.

In the UP-EGM group, the LSI values from the antero-superior and antero-inferior regions were greater than those from the postero-superior and postero-inferior regions in the left CCLs (*p* < 0.001), and similar results were found in the right CCLs. All the LSI values in the UP-EGM group were less than those in the corresponding regions in the LSI group (all *p* < 0.001). In addition, the variation of the LSI values in the UP-EGM group was significantly greater than that in the LSI group in all CCL regions (Figure 3).

### 3.5. Follow-Up

**Figure 4:**
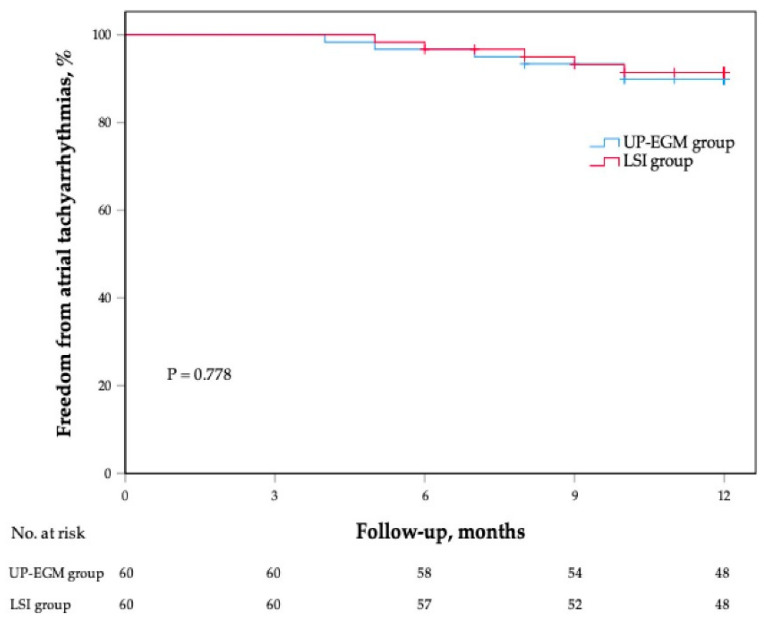
The Kaplan–Meier survival curve for atrial arrhythmia recurrence in both groups.

After a mean follow-up period of 11.31 ± 1.70 months, 54 patients (90%) in the UP-EGM group remained in sinus rhythm without AADs, whereas 55 patients (91.7%) in the LSI group remained in sinus rhythm (*p* = 0.752). The Kaplan–Meier survival curve demonstrated no significant difference in the sinus rhythm maintenance rate between the two groups (Figure 4). In the UP-EGM group, three patients (5%) presented with PAF recurrence, and three patients (5%) presented with AT recurrence. In the LSI group, three patients (5%) presented with PAF recurrence, and two patients (3.3%) presented with AT recurrence.

## 4. Discussion

### 4.1. Main Findings

The main findings of the present investigation were as follows: (1) The LSI values of catheter ablation guided by UP-EGM were less than the recommended values in all CCL regions. (2) The variation of the LSI values in the UP-EGM group was significantly greater than that in the LSI group in all CCL regions. (3) The procedural time, ablation time, and ablation energy delivered were significantly reduced by the catheter ablation guided by UP-EGM. (4) The first-pass PVI rate and 1-year SR maintenance rate were comparable between the UP-EGM group and the LSI group.

### 4.2. The LSI Values

The LSI as a novel guide for PVI was reported to be related to a lower rate of acute conduction gap formation and a higher single ablation success rate in previous studies [3,4,5,6,16]. However, the optimal LSI for successful PVI procedures is not clear [5]. In previous studies, Mattia L et al. suggested that an LSI range of 5.5–6 was pursued in the anterior and septal portions of the pulmonary vein antra (PVA) and was 5–5.5 elsewhere [3]. Sundaram S et al. chose an LSI value of 5.0 in the posterior locations, 5.5 in the anterior locations, and 6.0 in the region between the left atrial appendage and left superior pulmonary vein ridge [4]. In the study by Dello Russo A et al., it was reported an LSI mean value that is higher than 5.3 can be considered a good predictor of AF freedom at the 1-year follow-up [6]. Kanamori N et al. further evaluated the optimal LSI for anatomical PVI procedures and proposed that an LSI ≈ 5.2 may be a suitable target for effective lesion formation and that an LSI ≈ 4.0 may be acceptable in the posterior wall, especially in areas adjacent to the esophagus [5]. However, Cai C et al. chose target LSI values of 5.0 and 4.0 for the anterior and posterior walls in their study of the high-power radiofrequency catheter ablation of AF, and they also presented an LSI of 4.55 for the anterior wall and 3.95 for the posterior wall as the best cutoff values for predicting gap formation [16]. The rate of sinus rhythm maintenance ranged between 86% and 89.3% in previous investigations [3,4]. In our study, according to previous studies and the thickness of the atrial tissue in different regions of CCLs, we chose a target LSI value of 4.5–5.5 for the roof and anterior walls and 4.0–4.5 for the posterior and inferior walls. Our results in the LSI group showed that the actual mean LSI value was 4.5–5.5 for the roof and anterior walls and 4.0–4.5 for the posterior and inferior walls, and the first-pass PVI rate and 1-year SR maintenance rates were higher than or similar to those in prior studies, demonstrating that LSI was a useful guide for PVI [3,4,5,6,16]. However, the constant values of LSI do not take variations in underlying left atrial thickness into account [7,8]. Therefore, excessive ablation may occur in the posterior walls, which are adjacent to the esophagus.

### 4.3. The Relationship between UP-EGM and LSI

Previous studies have demonstrated that the elimination of the negative component of UP-EGM during radiofrequency applications reflects transmural lesions (TLs) and that unipolar signal modification is an effective guide for PVI [10,12]. All the recorded UP-EGMs turned completely positive in the UP-EGM group in the present study. This result is consistent with our previous study. Our previous study also showed that PVI was guided by UP-EGM with the above ablation setting; subsequently, the success rate was 86% after a mean follow-up of 19 ± 5 months [12]. The study of Pambrun T et al. demonstrated that high-power (40–50 W) PVI guided by UP-EGM was also safe and effective with the 90% sinus rhythm maintenance at 12 months [17]. In addition, the ablation duration for the elimination of the negative component of UP-EGM reflected the thickness of the ablation point [9,11]. The thicker the ablation tissue is, the more duration time that is needed for the elimination of the negative component of UP-EGM. Thus, the UP-EGM can automatically adjust according to variations in the left atrial thickness and can reflect whether the lesion deployed is presumed to be transmural in real time, and excessive ablation can be avoided in these lesions. We explored the actual LSI of the ablation tissue with initial irreversible transmurality according to the UP-EGM patterns. The LSI values from the antero-superior and antero-inferior regions were greater than those from the postero-superior and postero-inferior regions in the right and left CCLs (all *p* < 0.001), which is consistent with the anatomical site and atrial wall thickness [8]. All the LSI values in the UP-EGM group were less than those in the corresponding regions in the LSI group (all *p* < 0.001). Nevertheless, the first-pass PVI rate and 1-year SR maintenance rate in the UP-EGM group were comparable to those in the LSI group. These results demonstrate that the effectiveness of UP-EGM-guided radiofrequency catheter ablation in patients with PAF is not inferior to that of LSI and suggest that the LSI values may lead to excessive ablation.

### 4.4. The Potential Advantages UP-EGM

The previous study had demonstrated that TLs with a nonparallel or parallel catheter orientation consistently exhibited a complete abolition of the negative deflection with a mild attenuation of the positive deflection whereas that recorded from non-TLs did not [9]. Therefore, the elimination of the negative component on UP-EGM should not be influenced by the orientation of the catheter. This is the advantage of UP-EGM. Another concern is that the variation in the LSI values in the UP-EGM group was significantly greater than that in the LSI group in all CCL regions. This also indicated that the ablation guided by UP-EGM was individualized. In addition, the ablation time and the ablation energy delivered were significantly lower in the UP-EGM group than in the LSI group. However, the incidence and severity of esophageal injury in our study remain unknown due to the limited number of subjects. However, one patient (1.7%) developed reactive pneumonia, which was suspected to be related to the ablation in the LSI group. We think that ablation guided by UP-EGM may have safety implications by limiting the amount of RF energy delivered and (theoretically) reducing the risk of surrounding organ injury and LA perforation. However, this remains to be proven by more animal research and clinical trials.

### 4.5. Limitations

The current study had several limitations. First, this is a single-center investigation with a limited study sample. However, this was a prospective randomized study, and there was no significant difference in the baseline characteristics. Therefore, we believe the results of our study still have value as a clinical reference. Second, none of the patients with atrial tachycardia/AF recurrence in the UP-EGM group accepted a second PVI procedure. The mechanism of recurrence is unclear. Third, we had recorded the mean force value and the time of application from the beginning of application to complete unipolar electrogram for every ablation point in the UP-EGM group; however, the application time was also influenced by the left atrial thickness, which may differ in different regions. The relationship between contact force values and the time of application cannot be accurately analyzed in our study. Fourth, the follow-up lasted only one year. The results of the long-term follow-up should be further explored.

## 5. Conclusions

The unipolar electrogram and lesion index guided catheter ablation in patients with paroxysmal atrial fibrillation are both effective and safe. However, UP-EGM-guided catheter ablation may be more suitable than LSI for individual ablation.

## Figures and Tables

**Table 1:** Baseline characteristics.

Variables	UP-EGM Group (*n* = 60)	LSI Group (*n* = 60)	*p*-Value
Age, years	62.9 ± 10.3	62.5 ± 9.1	0.779
Male, *n* (%)	35 (58.3%)	29 (48.3%)	0.272
Body mass index, kg/m^2^	24.0 ± 3.0	24.0 ± 2.8	0.980
History of AF, months	24 (8.3, 60)	24 (9.5, 60)	0.694
CHA_2_DS_2_-VASc score	2 (1, 3)	2 (1, 3)	0.388
HAS-BLED score	1 (0, 2)	1 (0, 2)	0.547
Comorbidity			
Hypertension, *n* (%)	31 (51.7%)	38 (63.3%)	0.196
Diabetes mellitus, *n* (%)	7 (11.7%)	7 (11.7%)	1.000
Congestive heart failure, *n* (%)	4 (6.7%)	5 (8.3%)	0.729
Coronary artery disease, *n* (%)	4 (6.7%)	3 (5.0%)	0.697
Previous TIA/stroke, *n* (%)	6 (10.0%)	7 (11.7%)	0.769
Echocardiography			
LA diameter, mm	37.9 ± 5.3	36.8 ± 4.9	0.207
LVEF, %	64.2 ± 5.5	65.1 ± 5.2	0.348

LA—Left atrial; LVEF—left ventricular ejection fraction.

**Table 2:** Index AF Ablation Procedures.

Variables	UP-EGM Group (*n* = 60)	LSI Group (*n* = 60)	*p*-Value
Total ablation points	86.1 ± 12.5	89.7 ± 10.6	0.087
Procedural duration, min	101 (86, 112)	113 (105, 117)	<0.001
X-ray exposure, min	4.6 (3.4, 5.5)	6.0 (4.2, 8.3)	0.008
Ablation time, min	21.1 ± 2.4	32.6 ± 3.9	<0.001
Ablation energy delivered, kJ	49.4 ± 5.7	57.8 ± 14.5	<0.001
Mean CF, g	11 (10, 12)	11 (10, 12)	0.568
Mean impedance decrease, Ω	12 (11, 13)	11.5 (10, 14)	0.736
Required targeting of the carina regions for PVI completion, *n* (%)	11 (18.3%)	12 (20%)	0.817
The first-pass PVI, *n* (%)	54 (90%)	55 (91.7%)	0.752
Acutely PVI, *n* (%)	100%	100%	1.000
Additional ablation after PVI			
Cavotricuspid isthmus ablation, *n* (%)	6 (10.0%)	5 (8.3%)	0.752
Superior vena cava isolation, *n* (%)	1 (1.7%)	1 (1.7%)	1.000
Complications			
Reactive pneumonia, *n* (%)	0 (0%)	1 (1.7%)	1.000
Pseudoaneurysm, *n* (%)	1 (1.7%)	0 (0%)	1.000

CF—contact force; PVI—pulmonary vein isolation; PV—pulmonary vein.

**Table 3:** LSI in different regions of continuous circular lesions (CCLs).

Regions	UP-EGM Group (*n* = 60)	LSI Group (*n* = 60)	*p*-Value
**Left CCLs**			
Roof	4.12 ± 0.37	5.07 ± 0.18	<0.001
Antero-superior	4.23 ± 0.35	5.12 ± 0.13	<0.001
Antero-inferior	4.20 ± 0.33	5.03 ± 0.13	<0.001
Postero-superior	3.90 ± 0.29	4.43 ± 0.10	<0.001
Postero-inferior	3.83 ± 0.29	4.39 ± 0.10	<0.001
Inferior	3.98 ± 0.30	4.45 ± 0.11	<0.001
**Right CCLs**			
Roof	4.13 ± 0.30	4.75 ± 0.15	<0.001
Antero-superior	4.19 ± 0.40	5.16 ± 0.15	<0.001
Antero-inferior	4.15 ± 0.32	5.06 ± 0.16	<0.001
Postero-superior	3.91 ± 0.24	4.45 ± 0.11	<0.001
Postero-inferior	3.89 ± 0.24	4.38 ± 0.10	<0.001
Inferior	3.89 ± 0.24	4.45 ± 0.08	<0.001

LSI—lesion size index.

## Data Availability

The data used to support the findings of this study are available from the corresponding author upon reasonable request.

## References

[1] January C.T., Wann L.S., Calkins H., Chen L.Y., Cigarroa J.E., Cleveland J.C., Ellinor P.T., Ezekowitz M.D., Field M.E., Furie K.L. (2019). 2019 AHA/ACC/HRS Focused Update of the 2014 AHA/ACC/HRS Guideline for the Management of Patients with Atrial Fibrillation: A Report of the American College of Cardiology/American Heart Association Task Force on Clinical Practice Guidelines and the Heart Rhythm Society in Collaboration With the Society of Thoracic Surgeons. Circulation.

[2] Calzolari V., De Mattia L., Indiani S., Crosato M., Furlanetto A., Licciardello C., Squasi P.A.M., Olivari Z. (2017). In Vitro Validation of the Lesion Size Index to Predict Lesion Width and Depth After Irrigated Radiofrequency Ablation in a Porcine Model. JACC Clin. Electrophysiol..

[3] Mattia L., Crosato M., Indiani S., Causin E., Licciardello C., Maria Squasi P.A., De Leo A., Calzolari V. (2018). Prospective Evaluation of Lesion Index-Guided Pulmonary Vein Isolation Technique in Patients with Paroxysmal Atrial Fibrillation: 1-year Follow-Up. J. Atr. Fibrillation..

[4] Sundaram S., Choe W., Jordan J.R., Boorman C., Mullins N., Davies A., Stucky A., Nath S. (2018). Two Year, Single Center Clinical Outcome After Catheter Ablation for Paroxysmal Atrial Fibrillation Guided by Lesion Index. J. Atr. Fibrillation..

[5] Kanamori N., Kato T., Sakagami S., Saeki T., Kato C., Kawai K., Chikata A., Takashima S.I., Murai H., Usui S. (2018). Optimal lesion size index to prevent conduction gap during pulmonary vein isolation. J. Cardiovasc. Electrophysiol..

[6] Dello Russo A., Fassini G.M., Casella M., Romanelli E., Pala S., Riva S., Catto V., Moltrasio M., Tundo F., Zucchetti M. (2019). Lesion index: A novel guide in the path of successful pulmonary vein isolation. J. Interv. Card. Electrophysiol..

[7] Suenari K., Nakano Y., Hirai Y., Ogi H., Oda N., Makita Y., Ueda S., Kajihara K., Tokuyama T., Motoda C. (2013). Left atrial thickness under the catheter ablation lines in patients with paroxysmal atrial fibrillation: Insights from 64-slice multidetector computed tomography. Heart Vessel..

[8] Wang Y., Zhou G., Chen S., Wei Y., Lu X., Xu J., Wu X., Liu S. (2021). Tailored ablation index for pulmonary vein isolation according to wall thickness within the ablation circle. Pacing. Clin. Electrophysiol..

[9] Otomo K., Uno K., Fujiwara H., Isobe M., Iesaka Y. (2010). Local unipolar and bipolar electrogram criteria for evaluating the transmurality of atrial ablation lesions at different catheter orientations relative to the endocardial surface. Heart Rhythm.

[10] Bortone A., Appetiti A., Bouzeman A., Maupas E., Ciobotaru V., Boulenc J.M., Pujadas-Berthault P., Rioux P. (2013). Unipolar signal modification as a guide for lesion creation during radiofrequency application in the left atrium: Prospective study in humans in the setting of paroxysmal atrial fibrillation catheter ablation. Circ. Arrhythm. Electrophysiol..

[11] Bortone A., Brault-Noble G., Appetiti A., Marijon E. (2015). Elimination of the negative component of the unipolar atrial electrogram as an in vivo marker of transmural lesion creation: Acute study in canines. Circ. Arrhythm. Electrophysiol..

[12] Fu G., He B., Wang B., Liu J., Yu Y., Du X., Feng M., Gao F., Jin H., Fang R. (2019). Unipolar electrogram-guided radiofrequency catheter ablation in paroxysmal atrial fibrillation: Electrogram patterns and outcomes. J. Interv. Card. Electrophysiol..

[13] Park C.I., Lehrmann H., Keyl C., Weber R., Schiebeling J., Allgeier J., Schurr P., Shah A., Neumann F.J., Arentz T. (2014). Mechanisms of pulmonary vein reconnection after radiofrequency ablation of atrial fibrillation: The deterministic role of contact force and interlesion distance. J. Cardiovasc. Electrophysiol..

[14] El Haddad M., Taghji P., Phlips T., Wolf M., Demolder A., Choudhury R., Knecht S., Vandekerckhove Y., Tavernier R., Nakagawa H. (2017). Determinants of Acute and Late Pulmonary Vein Reconnection in Contact Force-Guided Pulmonary Vein Isolation: Identifying the Weakest Link in the Ablation Chain. Circ. Arrhythm. Electrophysiol..

[15] Hussein A., Das M., Riva S., Morgan M., Ronayne C., Sahni A., Shaw M., Todd D., Hall M., Modi S. (2018). Use of Ablation Index-Guided Ablation Results in High Rates of Durable Pulmonary Vein Isolation and Freedom from Arrhythmia in Persistent Atrial Fibrillation Patients: The PRAISE Study Results. Circ. Arrhythm. Electrophysiol..

[16] Cai C., Wang J., Niu H.X., Chu J.M., Hua W., Zhang S., Yao Y. (2022). Optimal Lesion Size Index for Pulmonary Vein Isolation in High-Power Radiofrequency Catheter Ablation of Atrial Fibrillation. Front. Cardiovasc. Med..

[17] Pambrun T., Durand C., Constantin M., Masse A., Marra C., Meillet V., Haïssaguerre M., Jaïs P., Bortone A. (2019). High-Power (40–50 W) Radiofrequency Ablation Guided by Unipolar Signal Modification for Pulmonary Vein Isolation: Experimental Findings and Clinical Results. Circ. Arrhythm. Electrophysiol..

